# BbWor1, a Regulator of Morphological Transition, Is Involved in Conidium-Hypha Switching, Blastospore Propagation, and Virulence in Beauveria bassiana

**DOI:** 10.1128/spectrum.00203-21

**Published:** 2021-07-28

**Authors:** Lei Qiu, Tong-Sheng Zhang, Ji-Zheng Song, Jing Zhang, Ze Li, Juan-Juan Wang

**Affiliations:** a School of Biological Science and Technology, University of Jinan, Jinan, China; b State Key Laboratory of Biobased Material and Green Papermaking, Qilu University of Technology, Shandong Academy of Sciences, Jinan, China; Broad Institute

**Keywords:** *Beauveria bassiana*, Wor1, morphological transition, virulence, germination, conidia, blastospore

## Abstract

Morphological transition is an important adaptive mechanism in the host invasion process. Wor1 is a conserved fungal regulatory protein that controls the phenotypic switching and pathogenicity of Candida albicans. By modulating growth conditions, we simulated three models of Beauveria bassiana morphological transitions, including CTH (conidia to hyphae), HTC (hyphae to conidia), and BTB (blastospore to blastospore). Disruption of *BbWor1* (an ortholog of *Wor1*) resulted in a distinct reduction in the time required for conidial germination (CTH), a significant increase in hyphal growth, and a decrease in the yield of conidia (HTC), indicating that BbWor1 positively controls conidium production and negatively regulates hyphal growth in conidium-hypha switching. Moreover, Δ*BbWor1* prominently decreased blastospore yield, shortened the G_0_/G_1_ phase, and prolonged the G_2_/M phase under the BTB model. Importantly, BbWor1 contributed to conidium-hypha switching and blastospore propagation via different genetic pathways, and yeast one-hybrid testing demonstrated the necessity of BbWor1 to control the transcription of an allergen-like protein gene (BBA_02580) and a conidial wall protein gene (BBA_09998). Moreover, the dramatically weakened virulence of Δ*BbWor1* was examined by immersion and injection methods. Our findings indicate that BbWor1 is a vital participant in morphological transition and pathogenicity in entomopathogenic fungi.

**IMPORTANCE** As a well-known entomopathogenic fungus, Beauveria bassiana has a complex life cycle and involves transformations among single-cell conidia, blastospores, and filamentous hyphae. This study provides new insight into the regulation of the fungal cell morphological transitions by simulating three models. Our research identified BbWor1 as a core transcription factor of morphological differentiation that positively regulates the production of conidia and blastospores but negatively regulates hyphal growth. More importantly, BbWor1 affects fungal pathogenicity and the global transcription profiles within three models of growth stage transformation. The present study lays a foundation for the exploration of the transition mechanism of entomopathogenic fungi and provides material for the morphological study of fungi.

## INTRODUCTION

The capacity of pathogenic fungi for morphological changes extends through the entire life cycle (1). The morphological transitions are usually controlled by transcriptional regulators, which initiate expression of a series of genes based on time, space, or environmental signals (2, 3). Candida albicans can transform among multiple phenotypic forms to adapt to and reside on a variety of hosts (4). The transcription factor Wor1 is considered to be the master regulator of cell morphological transformation of fungal white-opaque states in this fungal pathogen (5, 6). Wor1 controls its own level of expression, forming a stable autoregulatory feedback loop to induce and maintain the opaque state (7–9). The disruption of *Wor1* prevents opaque cell formation, while ectopic expression of the gene translates all fungal cells into stable opaque cells (7).

Although the morphological transformation of white-opaque cells only occurs in C. albicans and in closely related fungi, the Wor1 transcription factor is conserved throughout the fungal kingdom (10). Ryp1, a homologue of the Wor1 protein in the human pathogenic fungus Histoplasma capsulatum, is crucial for temperature-dependent mycelia-to-yeast transition and virulence (11). In plant pathogenic fungi, the *ros1* (a homologue of *Wor1*) deletion strain locks Ustilago maydis development in the filamentous stage and inhibits spore formation (12). ZtWor1 in Zymoseptoria tritici is pivotal for the production of spores and pathogenesis, and the gene disruption strain produces an extensive, dense mycelial network accompanied by a large number of abnormally swollen cell structures (13). Deletion of *CfWor1* in Cladosporium fulvum results in loss of virulence and damage to the formation of sclerotium-like structures and conidia (14). These results demonstrate that homologous genes of *Wor1* have evolved divergently in various human and plant fungal pathogens.

As a well-known entomopathogenic fungus, Beauveria bassiana is not only widely used as a fungal insecticide in controlling pests but also serves as a model system to investigate the interaction between fungal development and the host (15, 16). Under natural conditions, B. bassiana undergoes a transition among three cell morphologies, including hyphae, conidia, and blastospores (3). The conidia germinate, generate hyphae, and then directly invade host cuticles to infect insects (17). After intruding into the host hemocoel, B. bassiana experiences a dimorphic change to generate blastospores, which consume the nutrients existing in the hemolymph (15, 18). Finally, B. bassiana penetrates host tissues and cuticles and forms conidia to begin a new cycle of infection (19). For B. bassiana, both BbMbp1 (a component of the MluI cell cycle box-binding complex) and BbGEL1 (a gelsolin) play crucial roles in changing morphology to affect the development of conidia and blastospores (2, 3). Moreover, BbMbp1 mediates different transcriptomes and directly controls the expression of one cell wall protein gene and integral membrane protein gene to adapt to aerial and submerged conditions (2).

In this study, the role of BbWor1 was characterized in three models of the morphological transition process, including CTH (conidia to hyphae), HTC (hyphae to conidia), and BTB (blastospore to blastospore) in B. bassiana by gene disruption, complementation methods, and comparative transcriptomics analysis.

## RESULTS

### Bioinformatics description of BbWor1 and generation of its mutant strains.

Wor1 is considered the master regulator of cell morphological transformation in C. albicans (5). Based on a BLAST search with the CaWor1 protein sequence, BBA_06411 (identity 57.14%; E value 5e-27) was characterized and named BbWor1. Phylogenetic tree analysis revealed that BbWor1 shared 40% to ∼61% identity with homologues in human pathogenic fungi, plant pathogenic fungi, and yeast (Fig. S1A in the supplemental material). *BbWor1* encodes a 469-amino acid protein with a GTI1_PAC2 domain (amino acids +11 to +180) in its N-terminal region (Fig. S1B). According to quantitative real-time PCR (qRT-PCR), the transcript level of *BbWor1* increased by 5.4- and 1.8-fold in the BTB and HTC models, respectively, in contrast to the CTH model (Fig. S1C).

To reveal the functions of *BbWor1* in B. bassiana, a partial gene fragment was substituted by the phosphinothricin resistance gene to generate the Δ*BbWor1* disruption strain. The *BbWor1* open reading frame and the corresponding promoter regions were integrated into the deletion mutant strain to select the Δ*BbWor1*/*BbWor1* complementation mutant strain. The mutant strains were successively confirmed via PCR and qRT-PCR (Fig. S1D and E).

### Influence of BbWor1 on fungal development in the three models.

**FIG 1:**
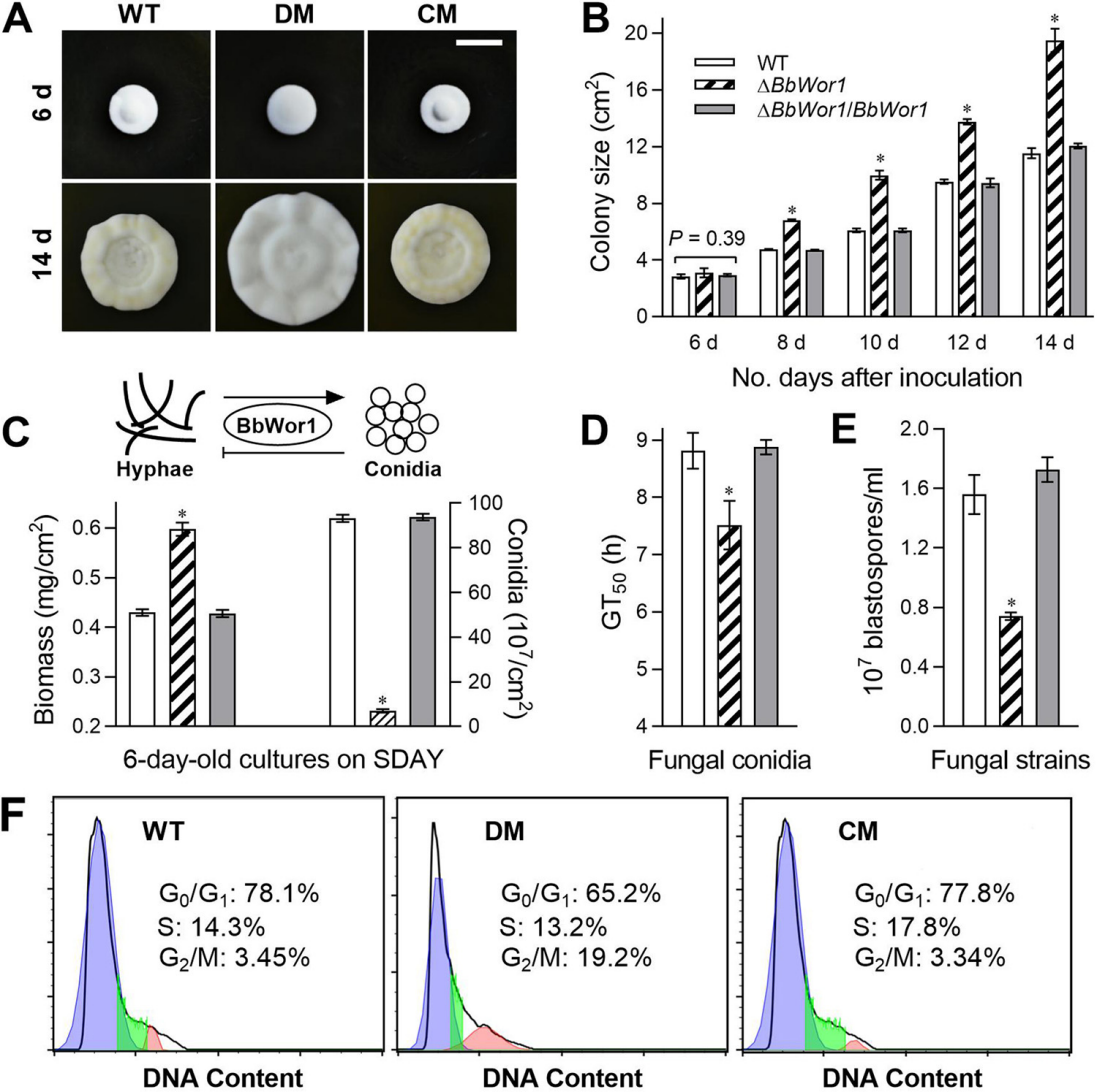
Disruption of *BbWor1* affects fungal vegetative growth and asexual development of B. bassiana. (A) Images of fungal colonies at 6 days and 14 days of growth under the temperature condition of 25°C on SDAY medium spotted with 1-μl aliquots of conidial suspension (scale bar: 20 mm). (B) Colony size of fungal colonies from 6 days to 14 days of culture on SDAY at 25°C. (C) Biomass and conidial yield of the fungal strains on SDAY plates for 6 days at 25°C in the HTC model (bottom). In the model of conidium-hypha switching by BbWor1 regulation, arrows and bars represent positive regulation and negative regulation, respectively (top). (D) Time until 50% conidial germination (GT_50_) in GB broth. (E) Blastospore yields after 4 days of culture in NLB medium at 25°C. (F) Cell cycle (G_0_/G_1_, S and G_2_/M phases) of unicellular blastospores determined by DNA content profiles with FACS analysis. The asterisk (*) denotes significant differences (Tukey’s HSD, *P < *0.05). Error bars, standard deviations (SDs) of three replicates.

The growth ability of fungal hyphae was measured by colony area comparison and biomass measurement. Colony area assays revealed no significant difference on day 6 postincubation on Sabouraud dextrose agar plates (SDAY) cultures between mutant strains and wild-type (WT) strains (*P > *0.05; Fig. 1A and B). However, the Δ*BbWor1* strain displayed obvious superiority in colony growth compared with the WT strain at 14 days (Fig. 1A) and was 1.4 to 1.7 times larger than the control strains (WT and complementation mutant) from 8 to 14 days (Fig. 1B).

Although no difference in colony area was observed at 6 days, the Δ*BbWor1* mutation biomass was increased by 18% compared with that of the WT in the HTC model (Fig. 1C). Moreover, the deletion strain produced 0.69 ± 0.11 × 10^8^ conidia/cm^2^ with an approximate 93% reduction compared with 9.32 ± 0.23 × 10^8^ conidia/cm^2^ for the WT strain and 9.38 ± 0.21 × 10^8^ conidia/cm^2^ for the complementation strain (Fig. 1C). In the CTH model, the Δ*BbWor1* strain showed an acceleration of approximately 15% in germination, with time to 50% germination (GT_50_) = 7.52 ± 0.24 h for the Δ*BbWor1* mutant compared with that of the control strains (Fig. 1D). In the CTH and HTC models, disruption of *BbWor1* significantly promoted the growth of hyphae and inhibited the production of conidia, indicating that BbWor1 positively regulates conidium production and negatively controls hyphal growth in conidium-hypha switching (Fig. 1C).

Blastospore incubation in nitrogen-limited broth (NLB) (BTB model) imitates the growth of B. bassiana in the hemolymph of insects. *BbWor1* disruption resulted in an approximately 55% decrease in blastospore yield. The Δ*BbWor1* mutant generated only 0.77 ± 0.02 × 10^8^ spores/ml, whereas the WT and complementation strains produced 1.68 ± 0.04 and 1.72 ± 0.05 × 10^8^ spores/ml, respectively (Fig. 1E). To explore the effect of *BbWor1* on the cell cycle in the BTB model, the DNA concentration from fluorescence-activated cell sorting (FACS) analysis was used to differentiate blastospores containing DNA-specific dye for the unduplicated (G_1_), duplicated (G_2_), and intermediate DNA concentration (S) profiles. The disruption mutant exhibited an altered cell cycle characterized by a shorter G_0_/G_1_ phase and longer G_2_/M phase compared with those of the control strains (Fig. 1F). Overall, the Δ*BbWor1* strain displayed significantly reduced spore production and a shortened G_0_/G_1_ phase in blastospores, but a prolonged G_2_/M phase.

### Effect of BbWor1 on fungal virulence.

**FIG 2:**
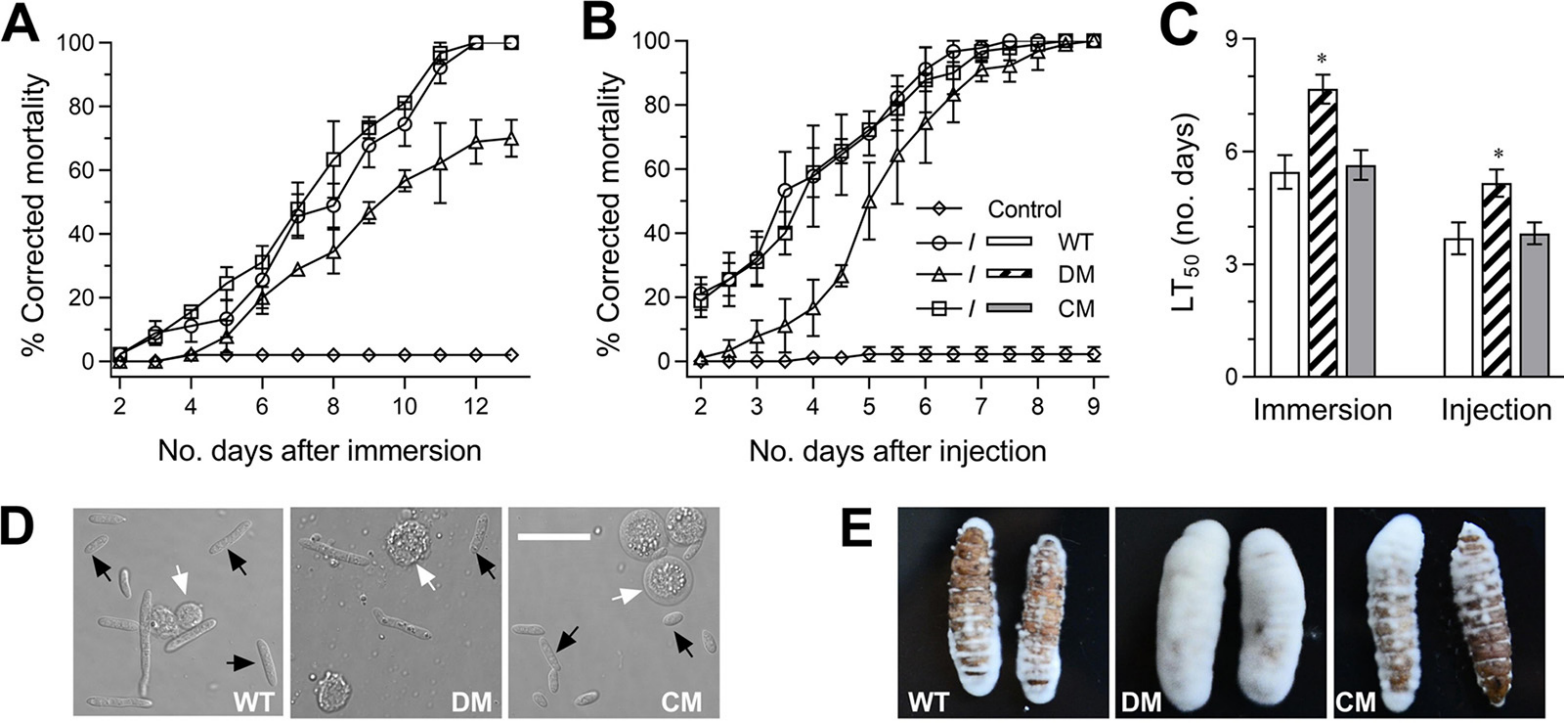
Effects of *BbWor1* loss on the virulence of B. bassiana. (A and B) Mortalities in immersion and intrahemoceol injection assays of Δ*BbWor1* (DM) and WT or Δ*BbWor1*/*BbWor1* (CM) were recorded. (C) The mean lethal time (LT_50_) for immersion and intrahemoceol injection tests were analyzed with Probit analysis. (D) Microscopy images (scale: 20 μm) obtained by LSCM for the blastospores in the hemolymph samples of surviving larvae at 3 days postinjection. Black arrows indicate blastospores, and white arrows indicate host hemocytes. (E) Images of fungal outgrowths at the surface of cadavers 3 days post-death through immersion (left) or injection (right) bioassays. The asterisk (*) denotes significant differences (Tukey’s HSD, *P < *0.05). Error bars, SDs of three replicates.

To explore how BbWor1 loss affects virulence, the lethality of B. bassiana against the moth Galleria mellonella was assayed to evaluate virulence as the median lethal time (LT_50_) by immersion or direct injection. Compared with the control strains, Δ*BbWor1* showed a dramatic decrease in mortality of insects infected by fungi (Fig. 2A and B). In immersion bioassays, the Δ*BbWor1* mutant strain displayed an LT_50_ of 7.67 ± 0.39 days, with LT_50_ = 5.46 ± 0.45 days for WT and LT_50_ = 5.64 ± 0.39 days for the complementation strain (Fig. 2C), demonstrating that disruption mutants require more time to kill insects than control strains. Similarly, direct injection bioassays displayed LT_50_ values of 5.16 ± 0.36 days for Δ*BbWor1*, 3.69 ± 0.43 days for WT, and 3.82 ± 0.28 days for the complementation strain (Fig. 2C). To further understand the cause of delayed lethal action, we examined hemolymph samples of live larvae that were cultured for 3 days following injection. Abundant blastospores were observed in the control strains; however, this number of individual cells was obviously reduced in the larvae infected with Δ*BbWor1* (Fig. 2D). In addition, after 3 days of maintenance under humid conditions, a large number of mycelia was observed outside of the cadavers killed by the Δ*BbWor1* mutant through the two infection modes, and the surfaces of cadavers killed by control strains were largely exposed (Fig. 2E). This phenomenon indicated that *BbWor1* deletion promoted the accumulation of B. bassiana hyphae on larval cadavers.

### Requirement of BbWor1 for global expression of the three models.

**FIG 3:**
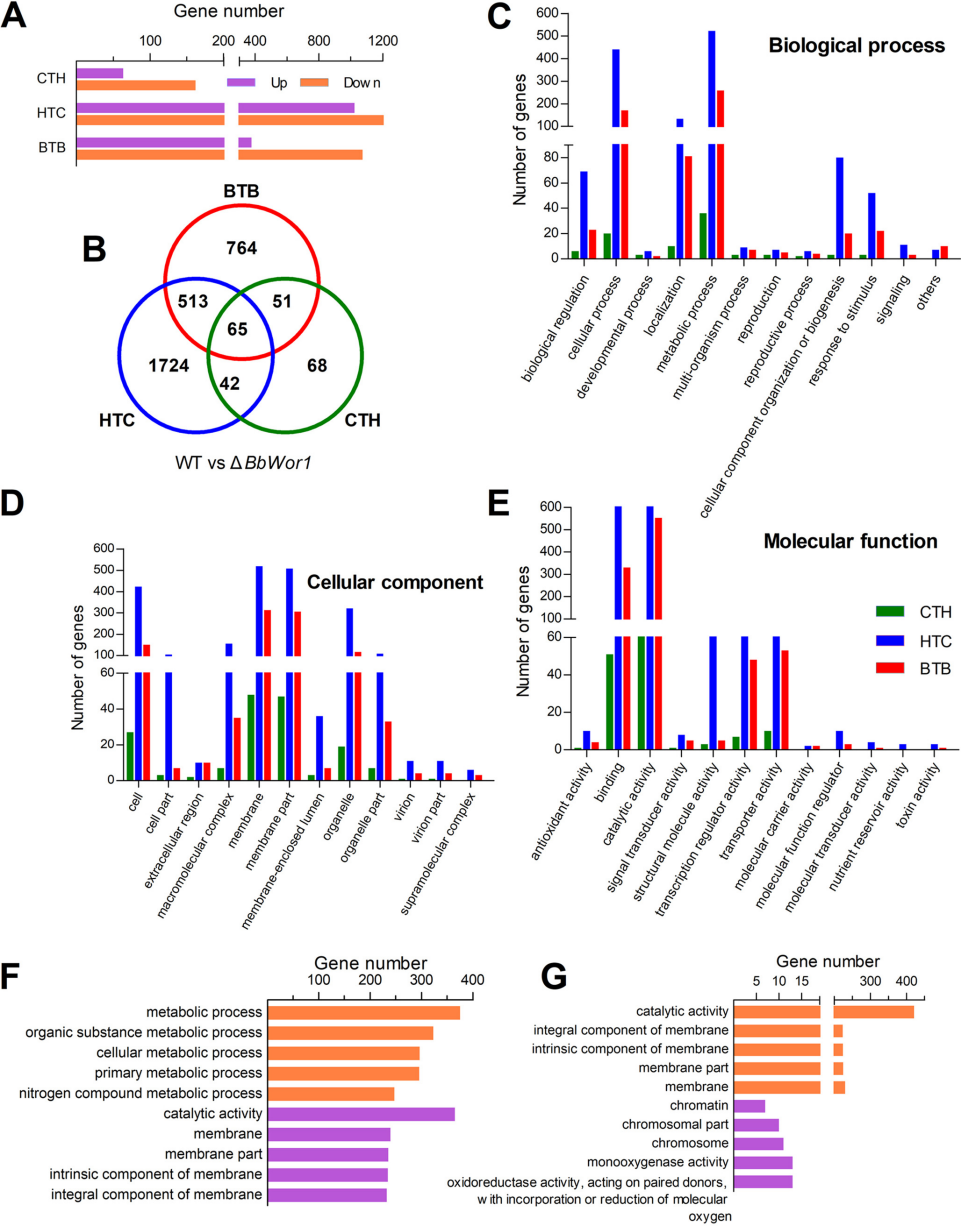
Disruption of *BbWor1* affects the transcription profile of B. bassiana in the three models. (A) DEGs whose expression was upregulated and downregulated were determined in the Δ*BbWor1* strain via comparison with WT in the CTH, HTC, and BTB models. (B) Venn diagram showing the number of DEGs between the WT and Δ*BbWor1* strains in the three models. (C to E) GO classification of DEGs into three main categories: biological processes, cellular components, and molecular functions. (F and G) GO enrichment analysis for DEGs whose expression was upregulated and downregulated between WT and Δ*BbWor1* in the HTC and BTB models. The top 5 terms are displayed (Q < 0.05).

According to the Cuffdiff method (20), differentially expressed genes (DEGs) (Q < 0.05, |log_2_FC| > 1) were screened between the WT and Δ*BbWor1* groups within three models of stage transformation simulated by modulated growth conditions. Compared with the BTB (1,393 DEGs) and HTC (2,344 DEGs) models, the CTH model had the fewest DEGs (226 genes) (Fig. 3A), indicating that disruption of *BbWor1* had the weakest effect on the number of genes in the CTH model. Further comparison analyses showed that most DEGs (578 genes) overlapped between the BTB and HTC libraries. Moreover, 107 DEGs overlapped between CTH and HTC, and 116 DEGs overlapped between CTH and BTB (Fig. 3B). Moreover, 65 DEGs overlapped among the three databases of the CTH, HTC and BTB models (Fig. 3B and Table S3).

Following gene ontology (GO) functional annotation analysis, the DEGs were concentrated in the three functional groups of biological processes, cell components, and molecular functions. As the GO terms were arranged in accordance with the number of genes, this suggested that the following top eight terms of the three models were the same: (i) cellular process and (ii) metabolic process in biological processes (Fig. 3C); (iii) cell, (iv) membrane, (v) membrane part, and (vi) organelle in cell components (Fig. 3D); and (vii) catalytic activity and (viii) binding in molecular functions (Fig. 3E).

However, despite that the top eight GO terms of DEGs were similar in the three models, gene expression regulated by BbWor1 within the three models was significantly different after GO enrichment. The DEGs in the CTH model were not significantly enriched in GO terms, suggesting that disruption of *BbWor1* does not significantly alter the type of genes expressed during germination. In the HTC model, genes whose expression was repressed were most enriched in metabolic processes (375 genes), and genes whose expression was upregulated were mostly enriched in catalytic activity (365 genes) (Fig. 3F; Table S4). Under the BTB model, catalytic activity (421 genes) was the enrichment term with the largest number of genes whose expression was repressed (Fig. 3G and Table S5). In addition, both the genes whose expression was upregulated in the HTC model and genes whose expression was repressed in the BTB model were collectively enriched in some terms, such as catalytic activity, integral component of membrane, intrinsic component of membrane, membrane part, and membrane (Fig. 3G). Such differences in GO enrichment indicated that *BbWor1* plays different roles in the three models.

### Interaction of BbWor1 with downstream genes.

**FIG 4:**
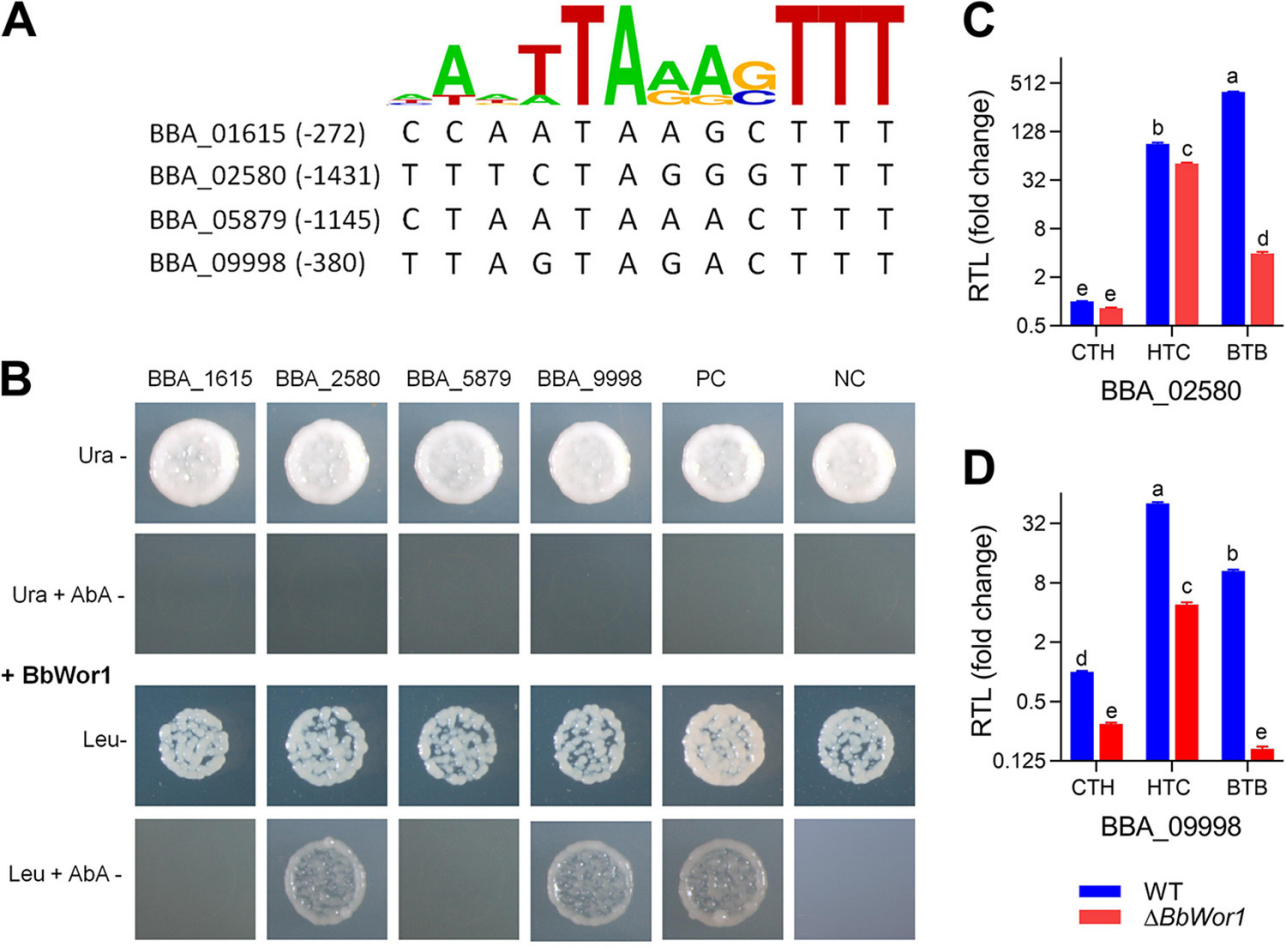
Identification of BbWor1 downstream genes and determination of the expression of target genes. (A) The binding site data of Wor1 (21, 22) were used to identify consensus binding sites in promoter regions of 4 genes selected from 37 common repressed DEGs in three models. (B) Yeast one-hybrid assay of the interaction of BbWor1 and 4 gene motifs. All motifs were introduced to autoactivation testing with 250 ng/ml aureobasidin A (AbA) on SD/−Ura medium, and physical interactions were measured on SD/−Leu medium with 250 ng/ml AbA. PC, positive control (yeast cells transformed with the pGADT7-Rec-p53 vector and p53-AbAi). NC, negative control (yeast cells transformed with pGADT7-Rec-BbWor1 and a blank vector [pAbAi]). (C and D) qRT-PCR validation of BBA_02580 and BBA_0998 expression. The relative transcript levels (RTLs) of the two genes are presented as the ratio of WT in the CTH model. Different lowercase letters denote significant differences (Tukey’s HSD, *P < *0.05).

To identify the potential BbWor1-mediated target genes, approximately 1.5 kbp upstream of each start codon (i.e., the promoter regions) of 37 genes that were repressed in all three models (Table S3) was screened in accordance with the binding motif of Wor1 (21, 22). Four genes (BBA_01615, BBA_02580, BBA_05879, and BBA_09998) were captured (Fig. 4A) and were considered potential targets of the BbWor1 protein for yeast one-hybrid experiments, in which it was demonstrated that BbWor1 directly mediated transcriptional activation of the BBA_02580 and BBA_09998 motif constructs incorporated into the reporter gene (Fig. 4B). BBA_02580 and BBA_09998 are allergen-like protein genes and conidial wall protein genes, respectively, on the basis of the content within the NCBI database. Compared with those in the WT under the CTH model, BBA_02580 and BBA_09998 transcript levels of the Δ*BbWor1* mutant were verified to be downregulated in all three models by qRT-PCR, especially in the BTB model, in which the levels decreased by 99% and 98%, respectively (Fig. 4C and D). The results from the qRT-PCR and the transcriptome sequencing (RNA-seq) data were consistent (Table 1), confirming the accuracy and quality of the transcriptome experiment.

**TABLE 1:** BbWor1-related genes described in this study and from the analysis of transcriptomes between Δ*BbWor1* and WT^*a*^

Locus tag	Annotation	CTH		HTC		BTB	
		Log_2_ FC	Q value	Log_2_ FC	Q value	Log_2_ FC	Q value
BBA_02580	allergen-like protein	−1.09	0.00	−5.46	0.00	−6.05	0.00
BBA_09998	conidial wall protein	−5.96	0.00	−3.87	0.00	−6.02	0.00
BBA_03082	β-1,3-glucanosyltransferase	1.08	0.00	2.34	0.00	−1.38	0.00
BBA_09307	acidic chitinase	1.43	0.00	0.11	0.00	−3.64	0.00
BBA_00047	Cel5b-like protein	1.52	0.00	−0.62	0.00	0.05	0.13
BBA_00048	hypothetical protein	1.14	0.00	−0.69	0.00	0.00	0.19
BBA_04942	fluG protein	0.34	0.28	−6.70	0.00	−1.39	0.00
BBA_02348	hsp70-like protein	−0.06	0.00	−1.29	0.00	0.11	0.00
BBA_00257	hsp70-like protein	−0.09	0.00	−1.12	0.00	0.06	0.00

a CTH, condia to hyphae; HTC, hyphae to condia; BTB, blastospore to blastospore; FC, fold change.

## DISCUSSION

As a well-known insect pathogenic filamentous fungus, B. bassiana is becoming a new typical fungus with which to investigate fungal development and pathogen-host interactions (15, 16). In the infection process, B. bassiana undergoes a series of morphological changes throughout the infection cycle, including filamentous hyphae, single-cell conidia, and blastospores. In the present study, we simulated three models of the morphological transition process of B. bassiana *in vitro*, including CTH, HTC, and BTB. After RNA-seq analysis of the three models with the WT strain was performed, the largest number of DEGs and the weakest correlation were found in the CTH and HTC models, indicating that the performance of B. bassiana in conidium-hypha switching differs more during gene transcription.

Wor1, a conserved fungal transcription factor, has been thoroughly studied in both human and plant pathogenic fungi. Wor1 is the master regulator of cell morphological transformation, especially in C. albicans (5, 7, 23). The change in fungal cellular states is accompanied by a change in the gene expression level of *Wor1* (6). The qRT-PCR results exhibited a notable discrepancy in *BbWor1* gene expression among the three simulation models, indicating that *BbWor1* may be related to the morphological transition of B. bassiana. In the present study, BbWor1 was highly influential in the control of vegetative growth and asexual development. Moreover, comparative transcriptomics demonstrated that BbWor1 participated in controlling the morphological transitions in the three models of B. bassiana via different genetic pathways.

Disruption of *BbWor1* reduced conidial germination time and promoted hyphal growth, possibly due to cell wall remodeling. Fungal β-1,3-glucanosyltransferases are glycosyl phosphatidylinositol-anchored proteins that affect cell wall biogenesis of fungi (24). In C. albicans, β-1,3-glucanosyltransferase (Phr1p) is a fungal cell wall remodeling enzyme conducive to hyphal plasticity and extension (25). The increased expression of BBA_03082 (which encodes a β-1,3-glucanosyltransferase) may accelerate the extension of the cell wall during conidial germination and hyphal growth (Table 1). The degradation by hydrolytic enzymes causes the cell wall to continue to relax and expand to release constraints on the cell wall to meet cell growth requirements (26–28). ChiE1 (chitinase) in Coprinopsis cinerea has been proven to participate in the extension and growth of stipe cell walls through breaking tethers and allowing chitin microfibrils to unclasp, thus increasing the space to add more polymers of chitin units and β-glucan subject to *in vivo* turgor pressure (29). In addition to upregulation of BBA_09307 (the homologous gene of *ChiE1*), the expression levels of two endoglucanase hydrolases (BBA_00047 and BBA_00048) were upregulated in CTH, which might account for the accelerated germination in the Δ*BbWor1* mutant, to some extent.

Conidia are important fungal cells for dispersal and persistence in the environment. B. bassiana grow saprophytically and produce conidia on cadavers and then enter the next stage of the infection cycle (2). In the HTC model, disruption of *BbWor1* significantly reduced (93%) conidial yield. FluG, as a conserved protein, plays vital roles in the conidiation process of filamentous fungi and is an upstream developmental activator in Aspergillus nidulans (30, 31). The expression level of BBA_04942 (the *FluG* homologous gene) significantly decreased (log_2_FC = −6.7) in Δ*BbWor1* mutant strains, suggesting there is an interaction between the Wor1-mediated pathway and the central regulatory pathway for conidiation. As a molecular chaperone in the endoplasmic reticulum (ER), LHS1 (from the heat shock protein 70 family) functions during protein translocation and protein folding in the ER, and loss of LHS1 severely impairs the conidiation of Magnaporthe oryzae (32). Expression of *Lhs1* homologous genes (BBA_02348 and BBA_00257) was repressed in the Δ*BbWor1* mutant, which might lead to a decrease in conidiation. The CTH and HTC models showed that BbWor1 positively controlled conidial production and negatively controlled hyphal growth by regulating different transcriptional maps in conidium-hypha switching.

Morphological transformation is critical for the pathogenesis of mycopathogens. The switch between opaque and white cells remarkably influences the interaction between C. albicans and the host. Opaque cells thrive in skin infections, while white cells are more frequently observed in internal infections (33). The disruption of *Wor1* maintains cells in the white state, which weakens the adaptability of C. albicans on the skin, thus affecting pathogenicity (5). In Fusarium oxysporum, deletion of *Sge1* not only is quantitatively involved in conidiogenesis, but also influences the expression of six effector proteins and reduces secondary metabolites, thereby reducing virulence (34). Disruption of *Fgp1* in Fusarium graminearum results in a lack of trichothecene toxin accumulation and greatly reduces virulence to wheat plants (35). ZtWor1, a transcriptional regulator of another wheat pathogen, plays a decisive role in the expression level of many genes encoding small secreted proteins and contributes to pathogenicity (13). In B. bassiana, blastospores consume insect hemolymph nutrients and/or result in symptoms caused by toxic metabolites from the pathogen (36). Therefore, the remarkable deterioration ability of the Δ*BbWor1* mutant for blastospore propagation might lead to significantly weakened fungal pathogenicity.

## MATERIALS AND METHODS

### Strains and growth conditions.

WT B. bassiana strain ARSEF 2860 was routinely fostered under a temperature of 25°C on Sabouraud dextrose agar plates (SDAY; 4% glucose, 1% yeast extract, 1% peptone, plus 1.5% agar) for 12 h in light and 12 h in dark. Escherichia coli strain DH5α (Shanghai, China) was amplified in Luria-Bertani (LB) broth. Agrobacterium tumefaciens strain AGL-1 was amplified in yeast extract broth (YEB; wt/vol: 0.5% sucrose, 0.1% yeast extract, 1% peptone, and 0.05% MgSO_4_) (37).

### Sequence analysis and generation of BbWor1.

The Wor1 protein sequence in C. albicans (XP_723567.2) was used as a query to search the B. bassiana genome (38) and a homologous protein was identified and appointed to BbWor1. BbWor1 was structurally compared with the protein sequences of human pathogens, phytopathogens, and yeasts, and the alignment was displayed in line with the ClustalW algorithm (39). A phylogenetic tree was constructed by MEGA 7 software (https://www.megasoftware.net/) with a neighbor-joining approach (40).

### Generation of gene deletion and complementation strains.

The *BbWor1* gene deletion and complementation vectors were constructed as described previously (41, 42). The primers used in this study are shown in Table S1 in the supplemental material. Briefly, a phosphinothricin resistance gene (*bar*) was used to replace a partial gene fragment (from −77 to +405) to form a gene deletion vector, and the entire *BbWor1* open reading frame, together with its corresponding promoter, was cloned into the complementation vector. The deletion and complementation vectors were transformed into WT and gene deletion mutant strains by the *Agrobacterium*-mediated transformation procedure. PCR and qRT-PCR were performed on potential transformants with primer pairs (Table S1) to verify correct recombination events.

### Assessments of fungal development in three models.

For the Δ*BbWor1* mutant, WT, and complementation strains, the diameters of fungal colonies inoculated by spotting of a 10^4^ conidial suspension (1 μl) were measured from 6 days to 14 days, and the colony areas on SDAY plates at 25°C were used as growth indices.

To explore morphological transitions in entomopathogenic fungi, we simulated the morphological transition of B. bassiana by three model sets of growth conditions. For the HTC model, aliquots (100 μl) of conidial suspension (10^7^ cells/ml) were plated on SDAY medium and cultured at 25°C for 6 days. The growth of mycelia was measured by drying fungal cultures overnight at 50°C and weighing them. The number of conidia per square centimeter was used to quantify the production ability of conidia. For the CTH model, fungal conidia cultured on SDAY were collected and added to 20 ml (10^6^ conidia/ml) of germination broth (GB; 2% sucrose and 0.5% peptone). The rate of germinating conidia was counted hourly after incubation for 6 h at 25°C (with aeration, 200 rpm). The median germination time (GT_50_, h), a viability indicator of 50% conidial germination, was computed following the fitted germination tendency of fungal conidia. For the BTB model, hyphae collected from SDAY medium were inoculated into NLB (4% glucose, 0.4% NH_4_NO_3_, 0.3% KH_2_PO_4_ and 0.3% MgSO_4_) to collect blastospores. As initial inocula, the resultant blastospores were adjusted to a final concentration of 10^4^/ml and then cultured for 4 days at 25°C in NLB to simulate a BTB model.

### Determination of the cell cycle.

Examination of the cell cycle for the Δ*BbWor1* mutant and control strains was carried out as mentioned above (43). Specifically, WT and mutant strain blastospore suspensions were treated with propidium iodide (DNA-specific stain) at a final concentration of 50 μg/ml at 4°C for 30 min. FACS analysis was performed on three 500-μl aliquots of every stained suspension (10^5^ conidia/ml) using a Beckman Coulter CytoFLEX LX flow cytometer for determination of DNA concentration. The G_0_/G_1_, G_2_/M, and S phases of the cell cycle were evaluated with the DNA concentrations.

### Insect bioassays.

The immersion and hemocoel injection methods were adopted to determine fungal virulence to G. mellonella (44). Specifically, a total of 30 larvae (∼300 mg each) were immersed in 30 ml of a conidial suspension (10^7^ conidia/ml) for approximately 10 s or injected into the abdomen with 1 μl of conidial suspension (5 × 10^5^ conidia/ml; 500 conidia per larva). The same volume of Tween 80 solution (0.02%) was used as a blank control. All batches of larvae were incubated in petri dishes (20 cm diameter) at room temperature at 25°C, and the mortality was examined at 12-h intervals. Probit analysis revealed the distinction between sigmoid time-mortality trends and median lethal time (LT_50_) estimates. The blastospores were observed in G. mellonella blood (45) using a laser scanning confocal microscope (Leica DMi8, Germany). The fungal hyphal growth on the humid cadaveric surface was recorded by taking photos.

### Extraction of RNA and comparative transcriptomics analysis of the three models.

To explore the relationship among the three models of WT and the role of BbWor1 in morphological transitions, comparative transcriptomics analysis was carried out among six treatments under the CTH (9 h), HTC (6 day), and BTB (4 day) models using WT and the Δ*BbWor1* mutant. Total RNA was extracted from fungal cultures of each mode with TRIzol-A^+^ reagent. Each treatment included three independent biological replicates. Sequencing of RNA samples was performed on the BGISEQ-500 platform at BGI-Shenzhen, China. Entire clean reads were mapped to the reference genome with HISAT2 (v2.0.4) (46). Bowtie2 (v2.2.5) (47) was applied to align the clean reads to the reference coding gene set, and the gene expression level was then evaluated by RSEM (v1.2.12) (48). The Cuffdiff method was used to analyze the differentially expressed genes (DEGs; Q value < 0.05, |log_2_ fold change (FC)| > 1) between the WT and disruption mutant strains (20). According to the gene ontology (GO) annotation results following the functional classification of DEGs, the phyper function in R software was used for enrichment analysis. Significant levels of terms and pathways were corrected by Q value (Q value < 0.05) with a Bonferroni correction (49).

### Screening of downstream genes and yeast one-hybrid test.

The yeast one-hybrid test was performed as previously described (2). The primer pair (Table S2) corresponding to different motifs was mixed, heated, and annealed by PCR to form double strands, which were then inserted into the pAbAi vector (Clontech) digested by HindIII and XhoI to generate the pBait-AbAi construct. The resulting construct was confirmed by AflII and XmaI digestion. Full-length *BbWor1* was amplified from cDNA with the p9/p10 primers (Table S2) and then cloned into the NdeI and EcoRI sites to construct the pGADT7-Rec-BbWor1 vector. The pGADT7-Rec-BbWor1 plasmid was transformed to a bait-specific reporter strain and then selected on appropriate selection plates (leucine-free SD medium with 250 ng/ml aureobasidin A; AbA). Transformation of yeast cells with the pGADT7-Rec-BbWor1 vector and one blank vector (pAbAi) was used as the negative control, while transformation of yeast cells with the p53-AbAi vector and pGADT7-Rec-p53 (Clontech) was used as the positive control. The positive colonies displayed an interaction of BbWor1 and the tested gene motifs.

### Gene expression analysis using qRT-PCR.

Total RNA samples were extracted and reverse transcribed into cDNA with TRIzol-A^+^ reagent (Tiangen Biotech, Beijing, China) and a FastKing RT kit (with gDNase) (Tiangen Biotech, Beijing, China). The 96 RT-PCR system (Thermo Fisher Scientific, USA) was used for quantitative real-time PCR (qRT-PCR) analysis with 2 × M5 HiPer SYBR Premix EsTaq (with Tli RNaseH) (Mei5 Biotechnology, Beijing, China) and the primers listed in Table S1. Fungal 18S rRNA was considered an endogenous standard, and the relative transcript levels of target genes were computed with the threshold cycle (2^-ΔΔCt^) method (50).

### Statistical analysis.

Entire phenotypic estimates from triplicate tests were acquired with one-way ANOVA. Tukey's honestly significantly difference (HSD) test was applied to determine the notable differences among fungal strains.

### Data availability.

RNA-seq data for this study are available at the NCBI GEO database (accession number GSE178974).
