# Supplementary material for: BbWor1, a Regulator of Morphological Transition, Is Involved in Conidium-Hypha Switching, Blastospore Propagation, and Virulence in Beauveria bassiana
Source: Microbiol Spectr. 2021 Jul 28;9(1):10.1128/spectrum.00203-21. doi: 10.1128/spectrum.00203-21 (PMC8552717; doi:10.1128/spectrum.00203-21)
Supplement: SUPPLEMENTAL FILE 1 — Supplemental material. Download SPECTRUM00203-21_Supp_1_seq7.pdf, PDF file, 0.5 MB [file spectrum00203-21_supp_1_seq7.pdf]

Supplemental Material FOR Publication

BbWor1, a regulator of morphological transition, is involved in conidium-hypha switching, blastospore propagation and virulence in *Beauveria bassiana*

Lei Qiu[a, b], Tong-Sheng Zhang[a, b], Ji-Zheng Song[b], Jing Zhang[b], Ze Li[b], Juan-Juan Wang[a]

[a]School of Biological Science and Technology, University of Jinan, Jinan, China

[b]State Key Laboratory of Biobased Material and Green Papermaking, Qilu University of Technology, Shandong Academy of Sciences, Jinan, China

[*]Corresponding author

***Juan-Juan Wang**: School of Biological Science and Technology, University of Jinan, Jinan, Shandong 250022, China. E-mail: wjj880414@163.com*

***Lei Qiu**: State Key Laboratory of Biobased Material and Green Papermaking, Qilu University of Technology, Shandong Academy of Sciences, Jinan, Shandong 250353, China. E-mail: qiulei.2005@163.com*

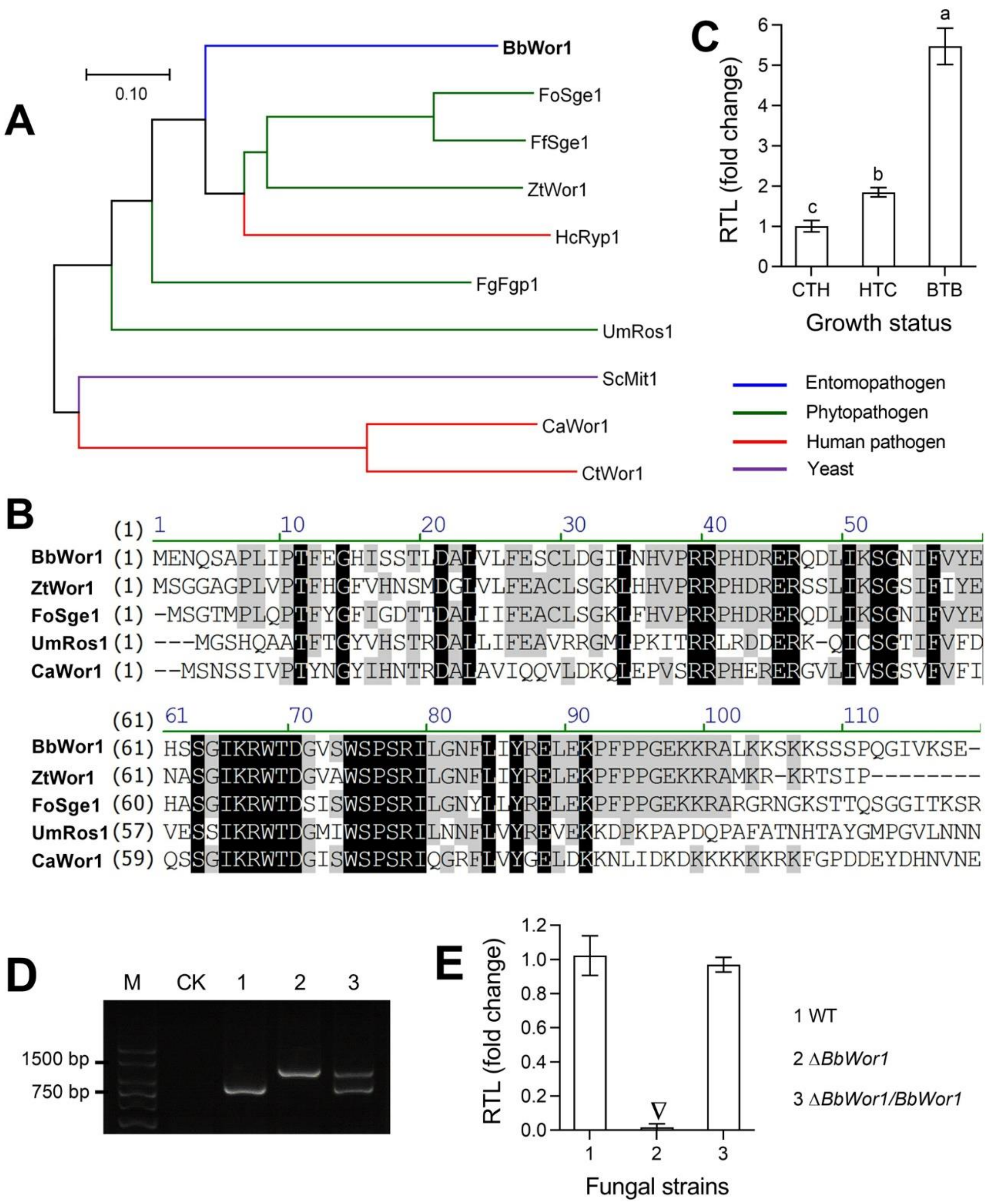

FIG S1 Bioinformatics description of *BbWor1* and generation of its mutant strain. A, Phylogenetic tree of BbWor1 with homologs from *Fusarium oxysporum* FoSge1, *Fusarium fujikuroi* FfSge1, *Zymoseptoria tritici* ZtWor1, *Histoplasma capsulatum* HcRyp1, *Fusarium graminearum* FgFgp1, *Ustilago maydis* UmRos1, *Saccharomyces cerevisiae* ScMit1, *Candida albicans* CaWor1 and *Candida tropicalis* CtWor1. The branch length represents the degree of sequence divergence. B, N-terminal region of the protein sequence alignment of BbWor1 with

Z. tritici ZtWor1, *F. oxysporum* FoSge1, *U. maydis* UmRos1 and *C. albicans* CaWor1. Conserved residues are marked in shaded black, and similar residues are marked in shaded gray. C, Relative transcript levels (RTLs) of *BbWor1* were determined by qRT-PCR and are presented as the ratio of the CTH group. D, Detection of *BbWor1* in WT (lane 1), Δ*BbWor1* (lane 2) and Δ*BbWor1/BbWor1* (lane 3) via PCR. E, qRT-PCR validation of the RTLs of *Bbwor1* in the WT, Δ*BbWor1* and Δ*BbWor1/BbWor1* strains. The RTL of *BbWor1* in each model is presented as the ratio of the CTH group according to the $2^{-\Delta\Delta Ct}$ method. The triangle pattern indicates that the fluorescence signal representing the intensity of gene expression has not been detected.

Table S1 The paired primers used for gene cloning, disruption, complement, and expression.

Primers	Paired sequences (5′-3′)#	Purpose
P1/P2	AAAGAATTCACCACCAATGTTGCTACCCC / AAACCCGGGGCTGAGAAGCTGGAAAAGTTAGAT	Cloning 5′ *BbWor1*
P3/P4	AAACTCGAGAGCAACAGCAAGGATCAGGA / AAAACTAGTTCAGGCACAGCAGTGGAGAC	Cloning 3′ *BbWor1*
P5/P6	GGGGACAAGTTTGTACAAAAAAGCAGGCTCGCCACGCTGGTCTATGTT / GGGGACCACTTTGTACAAGAAAGCTGGGTGTCGGATTAGACGAGGGATT	Cloning full-length *BbWor1*
P7/P8.	CAATTTGAAGACCGACAGGC / TACGAGTCCACGAGTGAGCC	PCR detecting *BbWor1*
P9/P10	CGGTAATATCTTCGTCTA / TCTGTAGATCAAGAAGTT	qPCR detecting *BbWor1*
P11/P12	TGGTTTCTAGGACCGCCGTAA / CCTTGGCAAATGCTTTCGC	qRT-PCR detecting 18S rRNA
P13/P14	TTCCAACTTCCTGTCCAA / CGGTGACGACTTCTTCAT	qRT-PCR detecting BBA_02580
P15/P16	CCTCTACCTCACCTTCAA / GGATCGTACTGGAATTGC	qRT-PCR detecting BBA_09998

Underlined regions denote the restriction enzyme sites for *BbWor1* disruption (*Eco*RI/*Xma*I and *Xho*I/*Spe*I) or the fragments of gateway exchange for targeted *BbWor1* complementation.

Table S2 The paired primers used for yeast one-hybrid to screen target genes.

Primers	Paired sequences (5′-3′)#	Purpose
p1/p2	AGCTTCCAATAAGCTTTCCAATAAGCTTTCCAATAAGCTTTC / TCGAGAAAGCTTATTGGAAAGCTTATTGGAAAGCTTATTGGA	Synthetic BBA_01615 motif
p3/p4	AGCTTTTTCTAGGGTTTTTTCTAGGGTTTTTTCTAGGGTTTC / TCGAGAAACCCTAGAAAAAACCCTAGAAAAAACCCTAGAAAA	Synthetic BBA_02580 motif
p5/p6	AGCTTCTAATAAACTTTCTAATAAACTTTCTAATAAACTTTC / TCGAGAAAGTTTATTAGAAAGTTTATTAGAAAGTTTATTAGA	Synthetic BBA_05879 motif
p7/p8	AGCTTTTAGTAGACTTTTTAGTAGACTTTTTAGTAGACTTTC / TCGAGAAAGTCTACTAAAAAGTCTACTAAAAAGTCTACTAAA	Synthetic BBA_09998 motif
p9/p10	CATATGATGGAGAATCAATCAGCGCCAT / GAATTCTCATGATCGGTGGAGAGAATTGG	Amplifying BbWor1 cDNA

Underlined regions denote the restriction enzyme sites to generate plasmid pGADT7-Rec-BbWor1 vector (*Nde*I and *Eco*RI).

Table S3 Expression levels of 65 DEGs overlapping in the three models in Δ*BbWor1 vs* WT.

Table S4 GO enrichment analysis was performed for DEGs whose expression was upregulated and downregulated between the WT *and* Δ*BbWor1* strains in the HTC model.

Table S5 GO enrichment analysis was performed for DEGs whose expression was upregulated and downregulated between the WT *and* Δ*BbWor1* strains in the BTB model.
